# Medical Schools: Bristol

**Published:** 1890-11-08

**Authors:** 


### Medical Schools.

### BRISTOL.

Most successful and enjoyable was the annual dinner of the Bristol Medical School which took place on Saturday, November 1st, at the Montague Hotel, Bristol, under the presidency of Mr. F. Richardson Cross, F.R.C.S. Nearly one hundred professors, medical practitioners, and students were present and the arrangements reflected great credit on Mr. J. Paul Bush, the Hon. Sec. After the usual loyal toasts,

Mr. Burdett proposed "The Bristol Medical School, the Royal Infirmary, and the General Hospital." He paid a well-deserved compliment to the authorities of the infirmary and hospital for the efficiency and *esprit de corps* which characterised the management of these institutions. He expressed the hope that the number of entries at Bristol would speedily increase as they ought to do, having regard to the eminence of several of the professors and teachers, and the excellence of the clinical work. He pointed out that the *Medical Annual* bore testimony to the ability and energy of many of the professors at Bristol, and that it was generally admitted that the *Medical Annual* was, on the whole, the best book of the kind published in the English language at the present time. It was desirable that Bristol should take a leaf out of the book of Manchester, Newcastle, and Birmingham, by erecting new school buildings and securing the early incorporation of the medical school with the college. Mr. Burdett then spoke at length upon certain aspects of medical relief, strongly urging the hospital authorities to ascertain what proportion the subscriptions of large firms bore to the amount of relief given to the employés sent by them for medical treatment in the course of each year. He advocated the reorganization of the out-patient department in the direction of evolving a system of weekly collections in the workshops, the proceeds to be placed in a box, from which the necessary funds should be taken from time to time to defray the cost of medical treatment by local practitioners for those of the employés who needed such aid. On the morning of Hospital Saturday the box should be opened, and all the money in it should be sent as a free gift to the medical institutions through the Hospital Saturday Fund. The hospitals should then make arrangements with the local medical practitioners to send on to the hospitals all the cases which came to them from the workshops which might prove of sufficient interest or importance to warrant their treatment by the staff. In this way the hospitals would become consulting-rooms for such of the patients of the general practitioners as needed special advice and aid.

Dr. E. Markham Skerritt, the Dean, replied for the Medical Schools. He said Bristol could hold its own with the London schools with regard to examinations, and it had not infrequently happened that Bristol students came out at the head of the list in the examinations of the London Boards. The clinical instruction at Bristol was so good that it would take a great deal to beat it. It was true that the entry during the present year was small, but that was one of those things that could no more be accounted for than they could account for its raining one day and being fine the next. That very week Mr. Greig Smith and himself had been fighting for the interests of the Provincial Schools. They had been to London in order to put before the Senate of the University of London the claims of the Provincial Medical Schools. They had good reason to hope that in any scheme for the reconstruction of the University of London, or for a new University, these schools would be allocated a good position. They had a very large out-patient department at Bristol, but he did not think that, on the whole, it was largely abused.

Dr. J. E. Shaw replied on behalf of the Royal Infirmary, and expressed his satisfaction at the visit of Mr. Burdett and the pleasure with which he had listened to his remarks as one to whom the construction and administration of hospitals were so intimately known. The Royal Infirmary was one of the oldest buildings now in use as a hospital, and its present high state of efficiency spoke well for the architect who designed it. The infirmary had made great progress; the patients sustained their numbers, the wards were more healthy; the nurses were highly trained and intelligent women, the surgeons were more bold than formerly, and last, but not least, the students were more numerous and infinitely more studious in the pursuit of their studies.

Dr. J. Michell-Clarke replied for the General Hospital. He referred to the friendly rivalry between the infirmary and the hospital, which led to a healthy competition, by which both institutions benefited in many ways. He welcomed back with great cordiality Mr. Penny, who had been absent for some time, owing to ill-health.

In replying to the toast of his health, Mr. Cross pointed out that there were a number of men springing up in Bristol at the present time who in ten or fifteen years would make their school one of the most flourishing in the country. In addition to the many excellent family practitioners, who were the backbone of the profession, and had been trained at Bristol, there were a number of young men on the staff who were determined to spread its reputation, to give the west of England confidence in its further development, and so to quicken the medical life of the country. He looked to the students to keep up the reputation of the Bristol school, and to do all they could to make themselves worthy of their *alma mater*.

